# GNS561 Exhibits Potent Antiviral Activity against SARS-CoV-2 through Autophagy Inhibition

**DOI:** 10.3390/v14010132

**Published:** 2022-01-12

**Authors:** Eloïne Bestion, Keivan Zandi, Sandrine Belouzard, Julien Andreani, Hubert Lepidi, Marie Novello, Clara Rouquairol, Jean-Pierre Baudoin, Madani Rachid, Bernard La Scola, Jean-Louis Mege, Jean Dubuisson, Raymond F. Schinazi, Soraya Mezouar, Philippe Halfon

**Affiliations:** 1 Genoscience Pharma, 13006 Marseille, France; eloine.bestion@gmail.com (E.B.); m.novello@genosciencepharma.com (M.N.); c.rouquairol@genosciencepharma.com (C.R.); m.rachid@genosciencepharma.com (M.R.); s.mezouar@genosciencepharma.com (S.M.); 2 Institut de Recherche Pour le Développement, Aix-Marseille University, Assitance Publique-Hopitaux de Marseille, Microbe, Phylogeny and Infection, 13005 Marseille, France; julien.andreani@univ-amu.fr (J.A.); jpbaudoin@live.fr (J.-P.B.); bernard.la-scola@univ-amu.fr (B.L.S.); jean-louis.mege@univ-amu.fr (J.-L.M.); 3 Institut Hospitalo-Universitaire Méditerranée Infection, Aix-Marseille University, 13005 Marseille, France; 4 Center for AIDS Research, Laboratory of Biochemical Pharmacology, Department of Pediatrics, Emory University School of Medicine, Atlanta, GA 30322, USA; keivan.zandi@emory.edu (K.Z.); rschina@emory.edu (R.F.S.); 5 Lille University, CNRS, INSERM, CHU Lille, Institut Pasteur de Lille, Centre d’Infection et d’Immunité de Lille (CIIL), INSERM U 1019—UMR 9017—UMR 8204, 59019 Lille, France; sandrine.belouzard@ibl.cnrs.fr (S.B.); jean.dubuisson@univ-lille.fr (J.D.); 6 Department of Pathology, La Timone Hospital, Aix-Marseille Université, 13005 Marseille, France; hubert.lepidi@ap-hm.fr

**Keywords:** GNS561/Ezurpimtrostat, SARS-CoV-2, COVID-19, autophagy, LC3

## Abstract

Since December 2019, SARS-CoV-2 has spread quickly worldwide, leading to more than 280 million confirmed cases, including over 5,000,000 deaths. Interestingly, coronaviruses were found to subvert and hijack autophagic process to allow their viral replication. Autophagy-modulating compounds thus rapidly emerged as an attractive strategy to fight SARS-CoV-2 infection, including the well-known chloroquine (CQ). Here, we investigated the antiviral activity and associated mechanism of GNS561/Ezurpimtrostat, a small lysosomotropic molecule inhibitor of late-stage autophagy. Interestingly, GNS561 exhibited antiviral activity of 6–40 nM depending on the viral strain considered, currently positioning it as the most powerful molecule investigated in SARS-CoV-2 infection. We then showed that GNS561 was located in lysosome-associated-membrane-protein-2-positive (LAMP2-positive) lysosomes, together with SARS-CoV-2. Moreover, GNS561 increased LC3-II spot size and caused the accumulation of autophagic vacuoles and the presence of multilamellar bodies, suggesting that GNS561 disrupted the autophagy mechanism. To confirm our findings, we used the K18-hACE2 mouse model and highlighted that GNS561 treatment led to a decline in SARS-CoV-2 virions in the lungs associated with a disruption of the autophagy pathway. Overall, our study highlights GNS561 as a powerful drug in the treatment of SARS-CoV-2 infection and supports the hypothesis that autophagy blockers could be an alternative strategy for COVID-19.

## 1. Introduction

In December 2019, severe acute respiratory syndrome coronavirus 2 (SARS-CoV-2) emerged to rapidly spread worldwide and was qualified as a pandemic [1]. As of October 2021, more than 280 million confirmed cases, including over 5,000,000 deaths, have been reported (World Health Organization data). The SARS-CoV-2-resulting disease, coronavirus disease 2019 (COVID-19), leads to a range of symptoms from a mild fever to acute respiratory distress syndrome, classifying this disease into several clinical categories, including asymptomatic, mild, moderate, severe, and critical infection [2]. The lack of effective curative strategies during the SARS-CoV-2 first wave has led to long-term hospitalizations and the submersion of health care systems. To address the urgent need for treatment options, numerous compounds were screened using SARS-CoV-2 cytopathic assays, including approved and investigational drugs. Among them can be cited remdesivir, for which the FDA granted emergency use authorization (EUA) [3]. Attributed EUA was nevertheless addressed to already hospitalized patients, aiming to prevent critical status and deaths. Orally available treatments that enable the prevention of general health status deterioration for less-severe patients are thus an imperative to hamper an actual sanitary crisis (who.int).

The diversion of the autophagy mechanism by viruses, including coronaviruses, allows viral replication [4]. More specifically, coronaviruses were found to benefit double-membrane vesicles (DMVs) to enhance the efficiency of virus replication [5]. SARS-CoV-2 was demonstrated to use DMVs as sites of viral RNA synthesis [6,7] and to interfere with autophagosome–lysosome fusion in vitro [8]. The use of autophagy inhibitors was therefore proposed to suppress SARS-CoV-2 replication. Chloroquine (CQ) and its derivative hydroxychloroquine (HCQ) first demonstrated promising SARS-CoV-2 antiviral effects in vitro [9,10,11,12]. However, these compounds were quickly overtaken by their ineffectiveness when tested in animal models [13,14]. More recently, additional data supported the idea that autophagy-inhibiting agents might be useful as therapeutic agents against SARS-CoV-2 infection in an in vivo model [15].

Our team developed an autophagy inhibitor, GNS561/Ezurpimtrostat, which is a lysosomotropic small basic lipophilic molecule that targets palmitoyl-protein thioesterase 1 (PPT1) [16] and induces lysosomal dysregulation, as proven by the inhibition of late-stage autophagy [17]. Currently under development in oncology indications (NCT03316222), GNS561 is being tested in a European clinical trial of patients infected by COVID-19 (NCT04333914). The objective of the current study was to evaluate the antiviral activity of GNS561 against SARS-CoV-2 infection. Here, we report that GNS561 has potent antiviral activity mediated by its ability to inhibit autophagic flux. These results open the prospect of using GNS561 as an innovative and effective treatment in coronavirus infections.

## 2. Materials and Methods

### 2.1. Reagents and Antibodies

Bafilomycin A1 (Baf A1, #B1793), cOmplete™ Protease Inhibitor Cocktail (#04693132001), and Fluoromount™ Aqueous Mounting Medium (#F4680) were obtained from Sigma–Aldrich (St Louis, MO, USA). Triton X-100 (#091584B) and Mammalian Cell Lysis Buffer (#28-9412-79) were provided by GE Healthcare (Chicago, IL, USA). Rabbit anti-light chain 3 phosphatidylethanolamine conjugate (LC3-II) (#2775, Cell Signaling Technology, Danvers, MA, USA), purified mouse anti-p62 (#610833, BD Biosciences, Franklin Lakes, NJ, USA), mouse antiglyceraldehyde-3-phosphate dehydrogenase (GAPDH) (#H00002597, Abnova, Taipei, Neihu, Taïwan) were used for Western blotting, and goat anti-mouse (#115-035-003) and goat anti-rabbit (#111-035-003) antibodies were purchased from Jackson ImmunoResearch (Newmarket, UK). For immunofluorescence assays, anti-LC3-II (#2775, Cell Signaling Technology), anti-LAMP2 (#AB_528129, clone H4B4, Developmental Studies Hybridoma Bank, Iowa City, IA, USA), anti-SARS/SARS-CoV-2 Coronavirus Spike Protein (subunit 1) (#PA5-81795, Invitrogen, Carlsbad, CA, USA), Alexa Fluor™ 647 Phalloidin (#A22287, Invitrogen), 4′,6-diamidino-2-phenylindole (#D1306, Invitrogen), Alexa 594-labeled anti-rabbit secondary antibody (#A21207, Invitrogen), Alexa 546-labeled anti-rabbit secondary antibody (#A11010, Invitrogen), Alexa 546-labeled anti-mouse secondary antibody (#A11003, Invitrogen), and Alexa 555-labeled anti-mouse primary and secondary antibodies (#A21424, Invitrogen) were used. Anti-SARS-CoV/SARS-CoV-2 Spike Protein S2 (#MA5-35946, Invitrogen), anti-ACE2 (#ab15348, Abcam), and 4’,6-diamidino-2-phenylindole (#D1306, Invitrogen) antibodies were used for immunohistochemistry experiments. For Western blot assays, N protein was detected using a rabbit polyclonal antibody (#NB100-56683, Novus Biologicals, Centennial, PA, USA) and a horseradish peroxidase-conjugated secondary antibody (#111-035-144, Jackson ImmunoResearch). Detection was carried out by chemoluminescence using Pierce™ ECL Western Blotting Substrate (#32209, Thermo Fisher Scientific, Cleveland, OH, USA).

### 2.2. Biosafety

Work with SARS-CoV-2 strains was performed in the biosafety level 3 laboratory of the IHU Mediterranean Infection (Marseille, France) and in the biosafety level 3 laboratory of the Center for Immunophenomics (CIPHE) for in vitro and animal experimentations, respectively.

### 2.3. Cell and Virus Culture

Vero E6 (African green monkey kidney, American Type Culture Collection, ATCC^®^ CRL-1586™) and Calu-3 (Human lung adenocarcinoma, ATCC^®^ HTB-55™) cells were cultured using Minimum Essential Media (MEM, Life Technologies, Carlsbad, CA, USA) supplemented with 10 and 15% fetal bovine serum (FBS, HyClone, Logan, UT, USA), respectively. Both media were supplemented with 1% penicillin–streptomycin (Pan Biotech, Aidenbach, Germany). Vero E6 medium was completed with 1% l-glutamine (#25030-024, Life Technologies). Vero E6-TMPRSS2 cells were grown in Dulbecco’s Modified Eagle Medium (DMEM, Gibco, Amarillo, TX, USA) supplemented with 10% heat-inactivated FBS (#10099141, Gibco). Cells were maintained at 37 °C in the presence of 5% CO_2_ and 95% air in a humidified incubator.

Vero E6-TMPRSS2 cell transfection was ensured by lentiviral vectors expressing TMPRSS2 that were produced by cotransfection of HEK-293T cells with a plasmid encoding HIV GagPol, a plasmid encoding VSVG and pTRIP-TMPRSS2. Cells were transfected using Turbofect (#R0531, Life Technologies) according to the manufacturer’s instructions. Supernatants were collected 48 h later and used to transduce Vero E6 cells and were then infected with SARS-CoV-2.

SARS-CoV-2 strains IHUMI-6, USA-WA1/2020, BetaCoV/France/IDF0372/2020, and hCoV-19_IPL_France (NCBI MW575140) were provided by the IHU Mediterranean Infection (Marseille, France), BEI Resources (Manassas, VA, USA), CIPHE (Marseille, France), and NCBI (Bethesda, MD, USA), respectively. All viral strains were previously propagated in the Vero E6 cell line and titrated by the tissue culture infectious dose 50 (TCID50) method followed by storage of aliquots at −80 °C until further use in the experiments.

### 2.4. Mice Model and Treatment

Female K18-hACE C57BL/6J mice (strain: 2B6.Cg-Tg (K18-ACE2)2Prlmn/J, 8-9 weeks) were obtained from Jackson laboratories and bred and housed in a ventilated cage system under SOPF requirements at the CIPHE animal facility (Parc scientifique et technologique de Luminy, CIML, Marseille, France) with standard chow diets. Randomized mouse females were treated for 24 h with 50 mg/kg GNS561, 200 µL per os (i.e., oral gavage administration), or with vehicle for the control group, before intranasal infection with 1.1 × 105 plaque-forming units (PFUs) of the BetaCoV/France/IDF0372/2020 SARS-CoV-2 strain in a final volume of 30 μL, as illustrated in Figure S2. Twenty-four hours after treatment, virus inoculation was performed under anesthesia that was induced and maintained with ketamine hydrochloride and xylazine, and all efforts were made to minimize animal suffering. Mice were then treated daily with GNS561 compound or vehicle. Mice were humanely euthanized at 7 days post-infection, and lung tissues were harvested.

### 2.5. In Vitro Cytotoxicity Assay

Cell viability was assessed using the CellTiter-Glo^®^ Luminescent Cell Viability Assay (#G7573, Promega, Madison, WI, USA) or using the CellTiter 96^®^ Non-Radioactive Cell Proliferation kit (#G4000, Promega) following the manufacturer’s instructions. Briefly, Vero E6 (25,000 cells/well) and Calu-3 (12,000 cells/well) cell lines were plated into a 96-well tissue culture plate with 175 µL of appropriate medium. Vero E6 and Calu-3 cells were treated for 2 h with 25 µL of increasing concentrations of tested drugs or with appropriate vehicle control and then infected with 50 µL of SARS-CoV-2 strain (IHUMI-6 or USA-WA1/2020) at 0.1 multiplicity of infection (MOI) and incubated for an additional 24 and 48 h, respectively. At the end of the treatment, CellTiter-Glo solution (IHUMI-6) or CellTiter 96^®^ Non-Radioactive solution (USA-WA1/2020) was added to each well. Cells were briefly shaken and then incubated at room temperature (RT) for 10 min to allow stabilization of the luminescent signal, which was recorded using an Infinite F200 Pro plate reader (Tecan, Männedorf, Switzerland). Cytotoxicity was expressed as the concentration of tested compounds that inhibited cell proliferation by 50% (CC_50_) and calculated using the Chou and Talalay method [18]. Every experiment was performed in technical triplicates, and three independent experiments were performed.

### 2.6. In Vitro Antiviral Activity Assays

Vero E6 (25,000 cells/well) and Calu-3 (12,000 cells/well) cell lines were plated into a 96-well tissue culture plate with 175 µL of appropriate medium and were allowed to adhere for a minimum of 24 h until the culture reached 90% confluence. Vero E6 and Calu-3 cells were treated with 25 µL of the tested drugs or vehicle control for 2 h and then infected with SARS-CoV-2 at 0.1 MOI and incubated for an additional 24 or 48 h, respectively. Viral extraction was performed on cell culture supernatants using a Macherey-Nagel™ NucleoSpin™ ARN viral kit (#12781021, Fischer Scientific, Hampton, NH, USA). The yield of progeny virus production was assessed using a specific qRT–PCR operating with previously extracted viral RNA. Briefly, one-step qRT–PCR was conducted in a final volume of 25 μL containing 10 µL of extracted viral RNA and 15 µL of a mix containing the probe/primer mix targeting the E gene (Table S1) and Super Script master mix from the SuperScriptTM III PlatinumTM One-Step qRT–PCR Kit (#11732020, Life Technologies). Quantitative PCR measurement was performed using the Cobas z 480 PCR system (Roche, Bâle, Switzerland), and data were analyzed with LightCycler 480 SW 1.5 software according to the manufacturer’s instructions (Roche). A melting curve analysis was performed after amplification to verify the accuracy of the amplicon. The yield of progeny virus production was calculated as the relative expression of the E gene of the considered condition normalized to both infected and untreated condition. Every experiment was performed in technical triplicates, and three independent experiments were performed.

Vero E6 and Vero E6-TMPRSS2 cells were plated for 24 h in a 24-well plate containing complete medium. Cells were infected at an MOI of 0.025 with increasing concentrations of GNS561 or with vehicle and incubated for an additional 16 h with SARS-CoV-2 (hCoV-19_IPL_France strain). Cells were rinsed with phosphate-buffered saline (PBS) and lysed in non-reducing Laemmli loading buffer (#84788, Thermo Fisher Scientific). Proteins were then separated onto a 10% SDS-polyacrylamide gel and transferred to a nitrocellulose membrane. SARS-CoV-2 nucleocapsid (N) protein was detected using a rabbit polyclonal antibody (1:1000) following a horseradish peroxidase-conjugated secondary antibody (1:10,000). Detection was carried out by chemoluminescence, and image quantification was performed using ImageJ software (NIH, Bethesda, MD, USA). Experiments were performed in duplicate.

### 2.7. Mice Tissue Investigation

Mouse tissues were weighed and homogenized with ceramic beads in a tissue homogenizer instrument (Precellys, Bertin Instruments, Montigny-le-Bretonneux, France) in 0.5 mL of RPMI media (#11875085, Gibco) supplemented with 2% FBS and 0.5 mL of RLT buffer (#79216, QIAGEN, Valencia, CA, USA). Tissue homogenates were clarified by centrifugation and stored at −80 °C. For subgenomic viral titration by RT–qPCR, RNA was extracted using the RNeasy Mini Kit (#74106, QIAGEN) and reverse transcribed using the High-Capacity cDNA Reverse Transcription Kit (#4368814, Thermo Fisher Scientific). Amplification was carried out using ONEGreen Fast qPCR Premix (#OZYA008, Ozyme, Saint-Quentin-en-Yvelines, France) according to the manufacturer’s recommendations. Copies of the N gene in samples were determined using primers targeting the N1 region of the N gene (Table S1). Copies of SARS-CoV-2 were compared and quantified with a standard curve and normalized to mg of tissue or RNA levels.

### 2.8. Immunofluorescence Assays and Analysis

For cellular investigation, Vero E6 cells (500,000 cells/well) were cultured the day prior to infection in a 24-well plate containing a glass coverslip and 700 µL of medium. Cells were then treated with 100 µL of GNS561G, a fluorescent analog of GNS561, or vehicle for 2 h and thereafter infected by SARS-CoV-2 strain (IHUMI-6, MOI 0.1) for an additional 48 h. Infected cells were then rinsed with PBS and fixed with 3% paraformaldehyde for 20 min at 4 °C. Cells were washed again with PBS, and aspecific sites were blocked with blocking buffer (1× PBS, 5% FBS) for 25 min at RT, followed by a permeabilization step with 3% Triton^TM^ X-100 for an additional 5 min in blocking buffer. Next, the cells were incubated for 45 min at RT with antibodies directed against LC3B (1:200), LAMP2 (1:250), and SARS Spike protein (1:250) and with Phalloïdin-647 (1:250) and 4′,6-diamidino-2-phenylindole (DAPI, 1:250) to reveal actin filaments and nuclei, respectively. Coverslips were then washed three times with PBS and incubated for 30 min at RT with appropriate secondary antibodies (1:1000) diluted in blocking buffer, including Alexa 594-conjugated anti-rabbit secondary antibody, Alexa 555-conjugated anti-mouse secondary antibody or Alexa 546-conjugated anti-mouse secondary antibody, to reveal SARS-S-2 protein, LC3B, and LAMP2, respectively. Labeled cells were washed three times with PBS and then mounted using FluoromountTM Aqueous Mounting Medium and stored overnight at 4 °C before analysis.

For image analysis, fluorescence pictures were acquired using an ApoTome module associated with a Zeiss microscope (Zeiss, Germany) equipped with an AxioCam MRm camera and collected by AxioVision software (Zeiss, Germany) and with an LSM 800 Airyscan confocal microscope (Zeiss, Germany), both with a 63× oil objective. At least five randomly selected microscopic fields were acquired in biological triplicates using Zen 3.0 software (Blue Edition, Zeiss, Germany) for cell investigation. Labeled areas from selected fields were quantified using ImageJ software. In Vero E6 cells, LC3B clusters were defined as an accumulation of LC3 single puncta with a size greater than 0.5 µm. For cell investigation, three independent experiments were performed with at least 5 random fields acquired each.

### 2.9. Cell and Mice Organs Electron Microscopy Imaging

Vero E6 cells (2,100,000 cells) were cultured the day prior to infection in a T75 flask containing 7 mL of culture medium, reaching 90% confluency the day after. Cultivated cells were treated with 4 µM GNS561 or vehicle control for 2 h and thereafter infected for an additional 24 h with the SARS-CoV-2 strain (IHUMI-6, MOI 0.1). Infected cells were harvested and stored for at least 1 h with glutaraldehyde 2.5% in 0.1 M sodium cacodylate buffer, in the same way as lung tissue samples. For resin embedding, the samples were washed three times with a mixture of 0.2 M saccharose/0.1 M sodium cacodylate and then postfixed for 1 h with 1% OsO4 diluted in 0.2 M potassium hexa-cyanoferrate (III)/0.1 M sodium cacodylate solution for 10 min. Samples were washed with distilled water and gradually dehydrated with ethanol in successive 10 min baths in 30%, 50%, 70%, 96%, and 100% ethanol. Substitution was achieved by successively placing the samples in 25, 50, and 75% Epon solutions for 15 min and they were then placed for 1 h in 100% Epon solution and in fresh Epon 100% overnight for two days at RT. Polymerization occurred with the cells and mouse lungs in 100% fresh Epon for 72 h at 60 °C. Ultrathin 70 nm sections were realized using a UC7 ultramicrotome (Leica, Wetzlar, Germany) and placed on HR25 300 Mesh Copper/Rhodium grids (TAAB, Berks, England) before being contrasted according to the methods of Reynolds (40). Electron micrographs were obtained on a Morgagni 268D (Philips/FEI Company, Eindhoven, Netherlands) transmission electron microscope operated at 80 keV TEM for cell samples and on a Tecnai G2 TEM (Thermo Fisher Scientific /FEI) operated at 200 keV equipped with a 4096 × 4096 pixel resolution Eagle camera (FEI) for lung tissues. Quantification of the area of autophagic vacuoles was performed including autophagosomes, autolysosomes, and multilamellar bodies (MLBs). Image quantification was performed using ImageJ software.

### 2.10. Western Blot Assay

Vero E6 cells (2,000,000 cells) were cultured the day prior to infection in a T75 flask containing 7 mL of culture medium, reaching 90% confluency the day after. Cultivated cells were treated with GNS561 or vehicle control for 2 h and thereafter infected for an additional 24 h by SARS-CoV-2 strain (IHUMI-6, MOI 0.1) and incubated at 37 °C in the presence of 5% CO2 and 95% air in a humidified incubator. During the last 4 h, cells were treated or not with 200 nM Baf A1, an autophagy flux inhibitor [19]. Western blotting was performed using 10 µg of protein, primary antibodies incubated at 4 °C overnight, and secondary antibodies incubated for 1 h at RT. The LC3-II (1:3000), p62 (1:1000), and GAPDH (1:5000) protein expression levels were investigated in association with goat anti-rabbit (1:40,000) and goat anti-mouse (1:20,000 or 1:40,000) secondary antibodies, respectively. Western blotting was performed using Imager iBrightCL1000 (Thermo Fisher Scientific), and analysis was performed using ImageJ software. The autophagic flux was calculated as the ratio between the LC3-II level normalized against the GAPDH level (Norm LC3-II) with BafA1 and without BafA1. All the experiments were performed in triplicate.

### 2.11. Statistical Analysis

All statistical analyses were performed using GraphPad Prism 9.2.0 (GraphPad Software Inc., La Jolla, CA, USA). For small datasets (<5, such as Western blotting and real-time polymerase chain reaction (PCR) datasets), the median and 95% confidence interval were calculated and used to compare different groups. For datasets (>5) with normal distribution, means were compared using one-way ANOVA with Dunnett’s post hoc analysis. The parametric paired t-test was used to compare two paired groups of data with a normal distribution. The Mann–Whitney two-tailed test was used to compare two unpaired groups of data without normal distribution. Data are presented as the mean values ± standard deviation (SD), except for small datasets (<5, such as Western blotting and real-time PCR datasets), for which data are presented as median values surrounded by upper and lower confidence limits (95%). Each *p* value was adjusted to account for multiple comparisons. Statistical significance was defined as *p* values < 0.05.

## 3. Results

### 3.1. GNS561 Exhibits Strong Antiviral Activity against SARS-CoV-2 Replication

**Figure 1:**
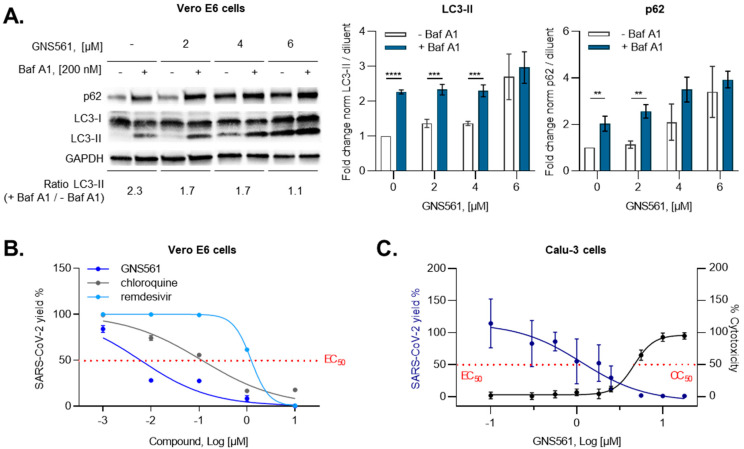
GNS561 antiviral activity. (**A**) GNS561 treatment blocks autophagic flux during SARS-CoV-2 infection. LC3-II and p62 protein expression was evaluated in uninfected Vero E6 cells treated with GNS561 by Western blotting and normalized to the GAPDH signal. Autophagy inhibition was evaluated in the presence (blue) or absence (white) of 200 mM bafilomycin A1 (Baf A1) treatment added 4 h before the end of the experiment. Representative autoradiograms are shown associated with the LC3-II (+ Baf A1/− Baf A1) ratio. Data values represent the mean ± SD from 3 independent experiments. Statistical analysis performed with unpaired t-test. *, *p* ≤ 0.05; **, *p* ≤ 0.01; ***, *p* ≤ 0.001; ****, *p* ≤ 0.0001. (**B**,**C**) GNS561 exhibits strong antiviral activity against SARS-CoV-2 replication. After 2 h of treatment with different compounds in the dose range, Vero E6 and Calu-3 cells were infected with SARS-CoV-2 strains for an additional 24 or 48 h, respectively. The viral yield was quantified and represented on the left axis of the graph. Values are the mean ± SD of at least triplicate assays conducted in triplicate. (**B**) The results obtained for Vero E6 cells treated with remdesivir, chloroquine, or GNS561 and infected with the USA-WA1/2020 strain are represented in light blue, gray, and dark blue, respectively. (**C**) The results obtained for the Calu-3 cell line treated with GNS561 and infected with the IHUMI-6 strain are represented with the half-maximal effective and cytotoxic concentrations on the left (blue line) and right (black line) axes on the graph.

To assess the antiviral activity of GNS561, we investigated SARS-CoV-2 copies using Vero E6 cells. First, regarding GNS561-specific effects on autophagy in the Vero E6 cell model, we performed a protein expression analysis focused on the LC3-II and p62 protein levels, two well-known autophagy markers [20]. Autophagy inhibition was evaluated at different drug concentrations applied for 24 h and in the presence or absence of bafilomycin A1 (Baf A1), a well-characterized inhibitor of the late stage of autophagy [19], added for the last 4 h of the experiment. Normalized LC3-II protein expression increased in a dose-dependent manner, without a further increase when Baf A1 treatment was added at the highest dose (Figure 1A), reflecting the accumulation of autophagosomes in cells. The same observation was made for the p62 protein level, suggesting that GNS561 blocks autophagic flux at the late stage in the Vero E6 cell line model.

Then, we evaluated the antiviral activity of GNS561 in a Vero E6 cell line model infected with the IHUMI-6 strain. As illustrated in Figure 1B and Table 1, GNS561 exhibited a 0.04 µM half-maximal effective concentration (EC_50_) with an 8.19 µM half-maximal cytotoxic concentration (CC_50_). Interestingly, the GNS561 compound showed the most potent antiviral effect against SARS-CoV-2 compared to CQ and remdesivir. Indeed, 0.26 µM EC_50_ (>200 µM CC_50_) and 2.02 µM EC_50_ (>50 µM CC_50_) were found for CQ and remdesivir, respectively. In an independent manner, GNS561 displayed 0.006 µM EC_50_ with 2 µM CC_50_, while CQ and remdesivir showed 0.1 µM EC_50_ (73.2 µM CC_50_) and 1.2 µM EC_50_ (>100 µM CC_50_), respectively, in Vero E6 cells infected with the USA-WA1/2020 SARS-CoV-2 strain (Figure 1B and Table 1). Overall, the GNS561 inhibitory concentration was 6.5–16.7 and 50.5–200 times higher than CQ and remdesivir according to the strain used. To confirm the antiviral activity of GNS561, we then used experimental models more suitable for the investigation of SARS-CoV-2 infection. In the human lung Calu-3 cell line, we reported that GNS561 antiviral activity was 1.1 µM EC_50_ (4.6 µM CC_50_) (Figure 1C), providing encouraging results for this compound as an efficient drug in SARS-CoV-2 infection.

### 3.2. GNS561 Disrupts the Autophagy Mechanism during SARS-CoV-2 Infection

**Figure 2:**
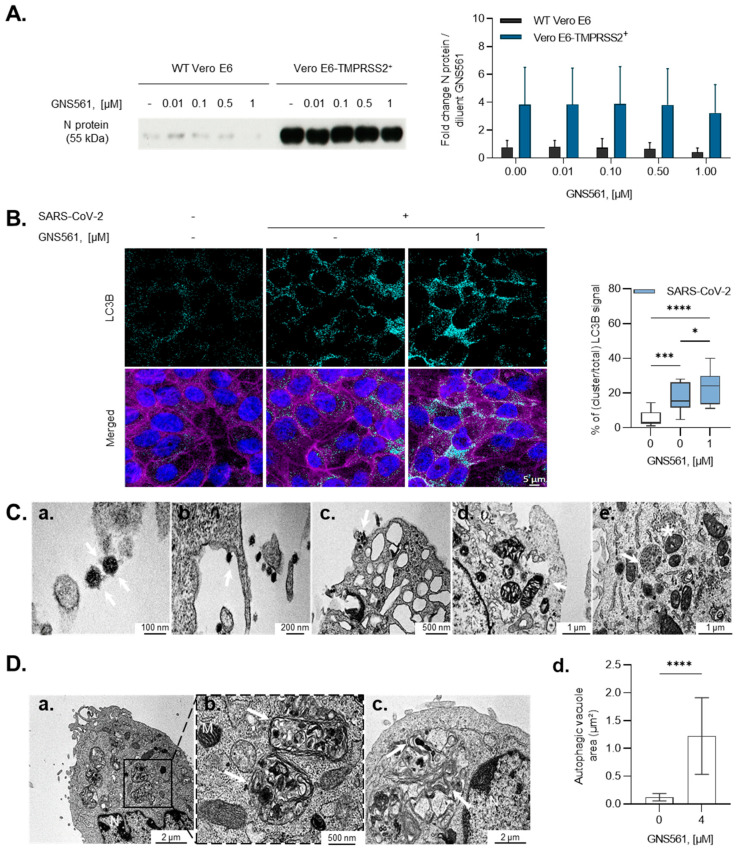
GNS561 mechanism of action during SARS-CoV-2 infection. (**A**) Antiviral activity of GNS561 during the entry step of the viral replication cycle was monitored using Western blot assay in the early time of infection. Wild-type (WT) and Vero E6-TMPRSS2+ cells were infected with the hCoV-19_IPL_France strain (NCBI MW575140) with increasing doses of GNS561. SARS-CoV-2 N protein quantification was conducted on cell lysates. Western blot quantification was performed in two independent experiments and is presented as the mean ± SD. (**B**) Immunofluorescence pictures acquired 48 h post-infection, illustrating LC3B protein presence (light blue) in uninfected/untreated condition (1B, left), infected condition (1B, middle), and infected/treated condition (1B, right). Nuclei and F-actin are represented in blue and purple, respectively. Superimposition of all channels is observable in the merged line. Quantification of LC3B clusters normalized to the total LC3B signal is represented in the right part of the figure panel. Values are representative of at least 5 random fields, conducted in triplicate from 3 independent experiments and expressed as the mean ± SD. Statistical analysis was performed with ordinary one-way ANOVA. *, *p* ≤ 0.05; **, *p* ≤ 0.01; ***, *p* ≤ 0.001; ****, *p* ≤ 0.0001. (**C**) Representative electron microscopy images of Vero E6 cells infected with the IHUMI-6 strain for 24 h. (**a**) The presence of the virus, (**b**) virus inking the cell surface, and (**c**) endocytic vesicles in the cytoplasm with clathrin-coated vesicles are indicated using white arrows. (**d**) Vacuole filled with nascent particles is indicated by a white arrow, (**e**) associated with virus budding inside the cytoplasm with a white asterisk. (**D**) Electron microscopy images of Vero E6 cells treated for 2 h with 4 µM GNS561 and then infected for an additional 24 h with the IHUMI-6 SARS-CoV-2 strain. (**a**,**b**) Autophagic vacuoles (AVs) exposing features of phagosomes/autophagosomes are indicated (white arrow), associated with (**c**) multilamellar bodies (MLBs) exposing features of lysosomal structures. (**d**) Quantification of the area of AVs is represented and expressed as the mean ± SD based on N ≥ 7 yields, with each yield accounting for one cell. N: nucleus, M: mitochondria. Statistical analysis performed with unpaired t-test. *, *p* = 0.05.

We next aimed to investigate the mechanisms associated with the antiviral activity of GNS561. Two key steps are currently targeted at the therapeutic level, the entry and replication of SARS-CoV-2. In the first series of experiments, we investigated the antiviral activity of GNS561 on the entry step of SARS-CoV-2 by the use of a TMPRSS2-expressing Vero E6 cell model, a key protease for the entry route of SARS-CoV-2 to infect host cells [21]. As illustrated in Figure 2A, GNS561 at increasing doses did not alter the N protein level compared to the untreated condition in either wild-type (WT) or Vero E6-TMPRSS2 cells, suggesting that GNS561 does not modulate either endocytosis entry or membrane fusion entry.

SARS-CoV-2 uses the autophagy machinery of host cells to promote its growth and replication [22,23,24,25]. Therefore, we next investigated the effects of GNS561, a lysosomotropic compound [17], during SARS-CoV-2 infection. First, we highlighted that both SARS-CoV-2 and GNS561 were localized to LAMP2-positive lysosomes (Figure S1). Then, we focused on the autophagy pathway by evaluating LC3B immunostaining. We observed that SARS-CoV-2 infection led to a significant increase in intracellular LC3B expression compared to the uninfected condition, as depicted in Figure 2B (*p* = 0.0004), suggesting that virus replication is dependent on the autophagy pathway. In addition, the LC3B signal was enhanced following supplemental GNS561 treatment, highlighting the modulatory properties of this compound on the autophagy pathway during SARS-CoV-2 replication (*p* = 0.03, Figure 2B). We then turned to the electron microscopy, which was particularly enlightening for the SARS-CoV-2 studies, allowing (1) the detection and the identification of SARS-CoV-2 virion adorned with its spike crown on its envelope (Figure 2Ca), (2) the anchorage and entry of SARS-CoV-2 at the periphery of cell membranes (Figure 2Cb,Cc), (3) the presence of endocytic vesicles in the cytoplasm with clathrin-coated vesicles containing amorphous material (Figure 2Cd), and (4) the virus-producing cell with vacuoles filled with nascent particles associated with the virus budding region inside the cytoplasm (Figure 2Ce). Electron microscopy moreover stands as the most accurate technique for both the detection and investigation of autophagy compartments [26,27]. We monitored that GNS561 treatment led to an increase in the volume of autophagic vacuoles in the cytoplasm of SARS-CoV-2-infected Vero E6 cells (Figure 2D). Indeed, we reported the presence of large and numerous cytoplasmic multilamellar bodies exposing features of lysosomal organelles containing multiple concentric membrane layers once Vero E6 cells were treated with GNS561 (Figure 2D).

**Figure 3:**
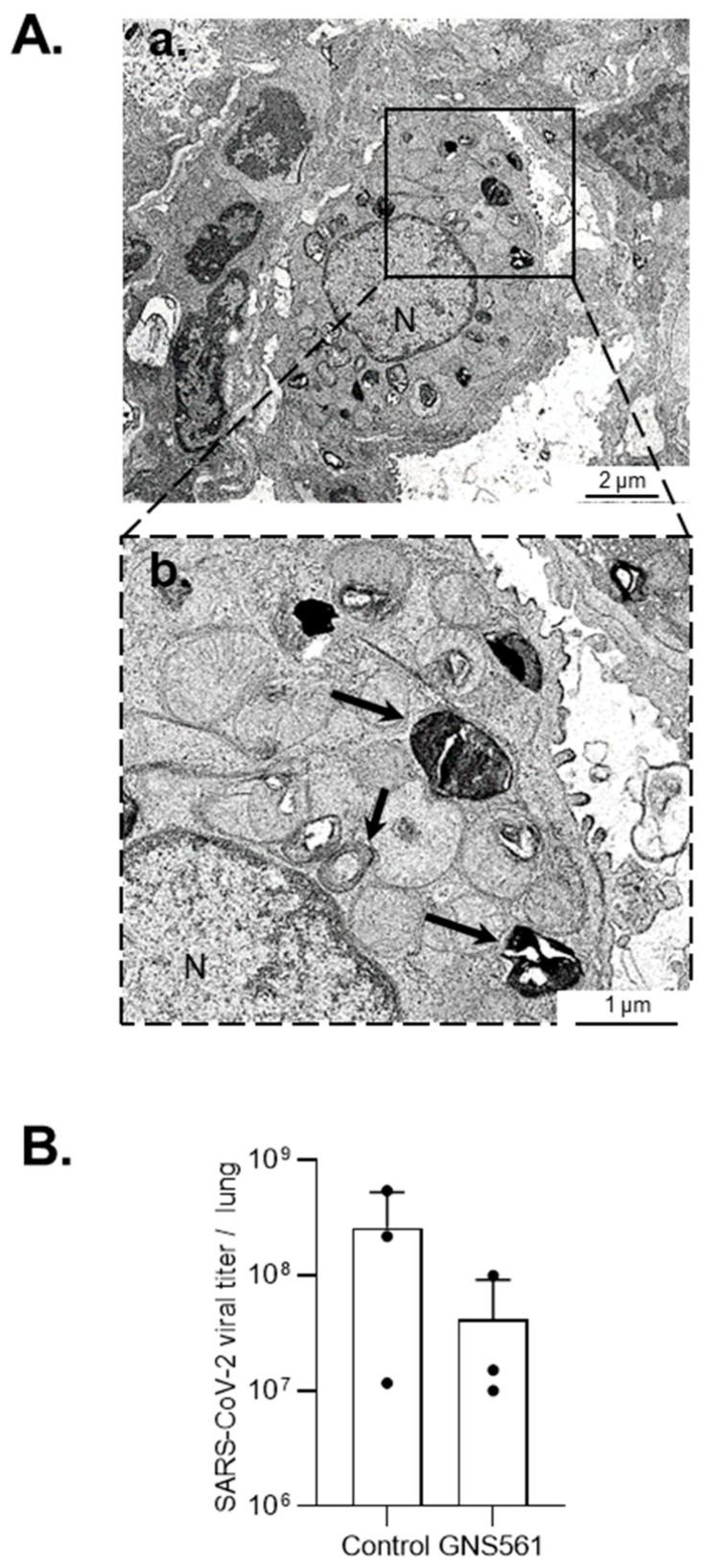
In vivo antiviral activity of GNS561. (**A**) Electron microscopy images of lung tissue from K18 hACE2 mice treated 24 h with GNS561 before SARS-CoV-2 infection and then treated daily with 50 mg/kg GNS561 compound and euthanized 7 days post-infection. Autophagic vacuoles are associated with MLBs (black arrow). (**B**) Lung viral titers from K18-hACE2 transgenic mice treated or not with GNS561 were investigated at 7 days post-infection by RT–qPCR. Three individuals were analyzed per group.

Finally, we investigated GNS561 antiviral activity in the K18-hACE2 mouse model (Figure S2). Ultra-structural analysis of lung tissue from K18-hACE2 mice infected with SARS-CoV-2 revealed autophagic vacuole accumulation with increased electron density (Figure 3Aa) and the presence of numerous cytoplasmic multilamellar bodies in GNS561-treated mice compared to untreated mice (Figure 3Ab). In vitro and in vivo accumulation and the increased size of the autophagic vacuoles suggested that lysosome-autophagosome fusion no longer occurred during GNS561 treatment of the SARS-CoV-2-infected model, leading to complex autophagy. Furthermore, a seven days post-infection analysis of the mouse lungs exhibited a downward trend of SARS-CoV-2 viral load in mice treated with GNS561 compared to the control group (*p* = 0.2410) (Figure 3B). Overall, our results indicate that the GNS561 autophagy inhibitor exhibits antiviral activity by impacting the autophagy mechanism during SARS-CoV-2 infection.

## 4. Discussion

In this work, we demonstrated that GNS561 exhibits a much stronger SARS-CoV-2 antiviral effect than CQ and remdesivir. Other autophagic flux inhibitors have been investigated to fight SARS-CoV-2 cytopathic effects. Interestingly, ROC-325 and clomipramine exhibited EC_50_ values of 3.28 μM and 13.6 μM, respectively, in the Vero E6 system without causing severe toxicity [28]. Gorshkov et al. indeed highlighted that a set of lysosome alkalizing small molecules were able to block SARS-CoV-2 cytopathic effects with EC_50_ values ranging from 2.0 to 13 μM, still in the Vero E6 model [28]. Among them were hycanthone, verteporfin, and mefloquine. To our knowledge, GNS561 thus exposes one of the most powerful in vitro antiviral effects against SARS-CoV-2 and is the only one developed in the clinic.

Our study additionally showed that GNS561 was located in LAMP2-positive lysosomes, similar to the SARS-CoV-2 S protein. Interestingly, Gosh et al. determined that mouse hepatitis virus and SARS-CoV-2, both members of the β-coronavirus family, exit cells via lysosomes rather than the biosynthetic secretory pathway more commonly used by other enveloped viruses [29]. As GNS561 colocalizes in the lysosomal compartment, the molecule could interfere with the proper egress process. It would therefore be interesting to explore GNS561’s effects on Rab7 and Arl8b expressions, as the authors showed that this unconventional discharge pathway is controlled by Arl8b-dependent lysosomal exocytosis. Moreover, the balance between Rab7 and Arl8b determines the subcellular localization of lysosomes [30], and their expression level variations can alter lysosome pH, for which strict control is primordial to guarantee virus release [31]. Always depicting SARS-CoV-2 egress, one of the preceding works demonstrated that GNS561 induces lysosomal membrane permeabilization [16]. It would thus be of interest to monitor lysosome structure impairment to provide supportive data regarding the observed strong decline in SARS-CoV-2 replication.

Our present work also demonstrated that GNS561 treatment provokes an accumulation and a size increase in autophagic structures in both in vitro and in vivo SARS-CoV-2-infected models. It is worth noting that the accumulation of unfused autophagosomes has been described during SARS-CoV-2 infection [32] and is regulated by the SARS-CoV-2 protein OFR3a. SARS-CoV-2 inhibition of autophagosome–lysosome fusion has been further validated by Zhang et al., who additionally showed that the ORF3a–VPS39 interaction prohibited the binding of the homotypic fusion and protein sorting (HOPS) complex with RAB7 [8]. The role of CQ in blocking autophagosome fusion has been proposed to be redundant with ORF3a function. It would thus be interesting to investigate the effects of both CQ and GNS561 on ORF3 and compare them to the previously described ORF3 regulation of autophagy during SARS-CoV-2 infection to dissect any difference explaining their distinct antiviral potency.

GNS561 is also known to significantly impair the enzymatic activity of cathepsins, including cathepsin L (CTSL) and cathepsin B (CTSB) [16,33]. Zaho et al. showed that functional CTSL is essential to cleave the SARS-CoV-2 S protein and enhance virus entry [34]. Another study demonstrated that both CTSB and TMPRSS2 are required for SARS-CoV-2 infection in ACE2-expressing human induced pluripotent stem (iPS) cells [35]. We also know that GNS561 does not block the SARS-CoV-2 entry pathway. We can thus hypothesize that the compound may affect late endocytic vacuoles regarding the entry process. Indeed, virions would succeed in entering host cells, but GNS561’s disruption of CTSB/L activity in the SARS-CoV-2 model could interfere with the fusion between the virus envelope and endosomal compartment, avoiding virus release into the cell cytoplasm and replicative cycle.

During the COVID-19 pandemic, drug investigations were led in reachable models at that point. They were thus at that time not necessarily the most relevant or suitable given the urgency of the situation. The use of in vitro models based on monolayer cultures of immortalized cell lines was proven to be fast and quite relevant [36,37]. In most of the available published papers, drug potency comparisons could be made using the Vero cell line model. However, it promptly appeared that human lung cell lines such as Calu-3 were more suitable models to confirm potential efficient antiviral activity [12,38]. GNS561 successfully passed the in vitro test, exposing an EC_50_ value of 1.1 µM in the Calu-3. The compound selectivity index is moderate and should be confirmed with more complex models such as cell cultures of human airway epithelial cells, including air-liquid interfaces or human airway organoids [36]. GNS561 was thereafter investigated in vivo using the K18-hACE2 mouse model. GNS561’s lung antiviral activity was shown to be moderate, with inter-individual variability tempering the compound degree of potency, although it strongly enabled neutralization in cells. An increase in the number of individuals or the use of a now-recognized more suitable in vivo model is to be expected.

Although CQ and HCQ present safety profiles in clinical use regarding indications for which they obtained market approvals, side effects from SARS-CoV-2 medication have been reported, including gastrointestinal upset [39], retinal toxicity [40,41], cardiomyopathy [42], and heart rhythm disturbances [43]. Compared to HCQ and CQ, GNS561 presents no phototoxicity and no electrocardiogram modifications, with markedly more potent activity against SARS-CoV-2 through its anti-autophagy mechanism. Interestingly, GNS561 is currently being tested in moderate COVID-19 patients in a phase II clinical study.

Taken together, due to the high potency of GNS561 against COVID-19 and its good safety profile observed in a phase I clinical trial [44,45], GNS561 constitutes a promising treatment for COVID-19.

## Figures and Tables

**Table 1:** Antiviral activity and cytotoxicity of GNS561, chloroquine, and remdesivir against SARS-CoV-2 in Vero E6 cells. After 2 h of drug treatment with different doses, Vero E6 cells were infected with SARS-CoV-2 virus, IHUMI-6 and/or USA-WA1/2020 strains (MOI 0.1), for 24 h. The half-maximal effective concentration (EC_50_) was investigated using qRT–PCR in µM. The half-maximal cytotoxic concentration (CC_50_), tested in the presence of the drug only, was assessed using a viability assay and addressed in µM.

SARS-CoV-2 Strain	IHUMI-6		USA-WA1/2020	
Drug [µM]	EC_50_	CC_50_	EC_50_	CC_50_
GNS561	0.04	8.19	0.006	2
Chloroquine	0.26	>200	0.1	73.23
Remdesivir	2.02	>50	1.2	>100

## Data Availability

The data presented in this study are available in the article or Supplementary Materials.

## Supplementary Materials

The following supporting information can be downloaded at https://www.mdpi.com/article/10.3390/v14010132/s1. Figure S1: SARS-CoV-2 and GNS561 localization inside lysosomes. Figure S2: Study design of the K18-hACE c57BL/6J mouse model. Table S1: Primer and probe sequences for SARS-CoV-2 E (cells) and N (mouse) gene q-RT-PCR investigation.

## References

[1] WHO Director-General’s Opening Remarks at the Media Briefing on COVID-19-11 March 2020. https://www.who.int/director-general/speeches/detail/who-director-general-s-opening-remarks-at-the-media-briefing-on-covid-19---11-march-2020.

[2] Verity R., Okell L.C., Dorigatti I., Winskill P., Whittaker C., Imai N., Cuomo-Dannenburg G., Thompson H., Walker P.G.T., Fu H. (2020). Estimates of the Severity of Coronavirus Disease 2019: A Model-Based Analysis. Lancet Infect. Dis..

[3] Commissioner O. FDA Approves First Treatment for COVID-19. https://www.fda.gov/news-events/press-announcements/fda-approves-first-treatment-covid-19.

[4] Choi Y., Bowman J.W., Jung J.U. (2018). Autophagy during Viral Infection—A Double-Edged Sword. Nat. Rev. Microbiol..

[5] Netherton C.L., Wileman T. (2011). Virus Factories, Double Membrane Vesicles and Viroplasm Generated in Animal Cells. Curr. Opin. Virol..

[6] Klein S., Cortese M., Winter S.L., Wachsmuth-Melm M., Neufeldt C.J., Cerikan B., Stanifer M.L., Boulant S., Bartenschlager R., Chlanda P. (2020). SARS-CoV-2 Structure and Replication Characterized by in Situ Cryo-Electron Tomography. Nat. Commun..

[7] Mohan J., Wollert T. (2021). Membrane Remodeling by SARS-CoV-2—Double-Enveloped Viral Replication. Fac. Rev..

[8] Zhang Y., Sun H., Pei R., Mao B., Zhao Z., Li H., Lin Y., Lu K. (2021). The SARS-CoV-2 Protein ORF3a Inhibits Fusion of Autophagosomes with Lysosomes. Cell Discov..

[9] Liu J., Cao R., Xu M., Wang X., Zhang H., Hu H., Li Y., Hu Z., Zhong W., Wang M. (2020). Hydroxychloroquine, a Less Toxic Derivative of Chloroquine, Is Effective in Inhibiting SARS-CoV-2 Infection in Vitro. Cell Discov..

[10] Wang M., Cao R., Zhang L., Yang X., Liu J., Xu M., Shi Z., Hu Z., Zhong W., Xiao G. (2020). Remdesivir and Chloroquine Effectively Inhibit the Recently Emerged Novel Coronavirus (2019-NCoV) in Vitro. Cell Res..

[11] Yao X., Ye F., Zhang M., Cui C., Huang B., Niu P., Liu X., Zhao L., Dong E., Song C. (2020). In Vitro Antiviral Activity and Projection of Optimized Dosing Design of Hydroxychloroquine for the Treatment of Severe Acute Respiratory Syndrome Coronavirus 2 (SARS-CoV-2). Clin. Infect. Dis..

[12] Hoffmann M., Mösbauer K., Hofmann-Winkler H., Kaul A., Kleine-Weber H., Krüger N., Gassen N.C., Müller M.A., Drosten C., Pöhlmann S. (2020). Chloroquine Does Not Inhibit Infection of Human Lung Cells with SARS-CoV-2. Nature.

[13] Rosenke K., Jarvis M.A., Feldmann F., Schwarz B., Okumura A., Lovaglio J., Saturday G., Hanley P.W., Meade-White K., Williamson B.N. (2020). Hydroxychloroquine Proves Ineffective in Hamsters and Macaques Infected with SARS-CoV-2. bioRxiv.

[14] Maisonnasse P., Guedj J., Contreras V., Behillil S., Solas C., Marlin R., Naninck T., Pizzorno A., Lemaitre J., Gonçalves A. (2020). Hydroxychloroquine Use against SARS-CoV-2 Infection in Non-Human Primates. Nature.

[15] Shang C., Zhuang X., Zhang H., Li Y., Zhu Y., Lu J., Ge C., Cong J., Li T., Li N. (2021). Inhibition of Autophagy Suppresses SARS-CoV-2 Replication and Ameliorates Pneumonia in HACE2 Transgenic Mice and Xenografted Human Lung Tissues. J. Virol..

[16] Brun S., Bestion E., Raymond E., Bassissi F., Jilkova Z.M., Mezouar S., Rachid M., Novello M., Tracz J., Hamaï A. (2021). GNS561, a Clinical-Stage PPT1 Inhibitor, Is Efficient against Hepatocellular Carcinoma via Modulation of Lysosomal Functions. Autophagy.

[17] Brun S., Bassissi F., Serdjebi C., Novello M., Tracz J., Autelitano F., Guillemot M., Fabre P., Courcambeck J., Ansaldi C. (2019). GNS561, a New Lysosomotropic Small Molecule, for the Treatment of Intrahepatic Cholangiocarcinoma. Investig. New Drugs.

[18] Chou T.-C. (2010). Drug Combination Studies and Their Synergy Quantification Using the Chou-Talalay Method. Cancer Res..

[19] Mauvezin C., Neufeld T.P. (2015). Bafilomycin A1 Disrupts Autophagic Flux by Inhibiting Both V-ATPase-Dependent Acidification and Ca-P60A/SERCA-Dependent Autophagosome-Lysosome Fusion. Autophagy.

[20] Klionsky D.J., Abdel-Aziz A.K., Abdelfatah S., Abdellatif M., Abdoli A., Abel S., Abeliovich H., Abildgaard M.H., Abudu Y.P., Acevedo-Arozena A. (2021). Guidelines for the Use and Interpretation of Assays for Monitoring Autophagy (4th Edition)^1^. Autophagy.

[21] Hoffmann M., Kleine-Weber H., Schroeder S., Krüger N., Herrler T., Erichsen S., Schiergens T.S., Herrler G., Wu N.-H., Nitsche A. (2020). SARS-CoV-2 Cell Entry Depends on ACE2 and TMPRSS2 and Is Blocked by a Clinically Proven Protease Inhibitor. Cell.

[22] Gassen N.C., Papies J., Bajaj T., Emanuel J., Dethloff F., Chua R.L., Trimpert J., Heinemann N., Niemeyer C., Weege F. (2021). SARS-CoV-2-Mediated Dysregulation of Metabolism and Autophagy Uncovers Host-Targeting Antivirals. Nat. Commun..

[23] Gorshkov K., Chen C.Z., Bostwick R., Rasmussen L., Xu M., Pradhan M., Tran B.N., Zhu W., Shamim K., Huang W. (2020). The SARS-CoV-2 Cytopathic Effect Is Blocked with Autophagy Modulators. bioRxiv.

[24] Gordon D.E., Jang G.M., Bouhaddou M., Xu J., Obernier K., White K.M., O’Meara M.J., Rezelj V.V., Guo J.Z., Swaney D.L. (2020). A SARS-CoV-2 Protein Interaction Map Reveals Targets for Drug Repurposing. Nature.

[25] Miao G., Zhao H., Li Y., Ji M., Chen Y., Shi Y., Bi Y., Wang P., Zhang H. (2021). ORF3a of the COVID-19 Virus SARS-CoV-2 Blocks HOPS Complex-Mediated Assembly of the SNARE Complex Required for Autolysosome Formation. Dev. Cell.

[26] Mizushima N. (2004). Methods for Monitoring Autophagy. Int. J. Biochem. Cell Biol..

[27] Swanlund J.M., Kregel K.C., Oberley T.D. (2010). Investigating Autophagy: Quantitative Morphometric Analysis Using Electron Microscopy. Autophagy.

[28] Gorshkov K., Chen C.Z., Bostwick R., Rasmussen L., Tran B.N., Cheng Y.-S., Xu M., Pradhan M., Henderson M., Zhu W. (2021). The SARS-CoV-2 Cytopathic Effect Is Blocked by Lysosome Alkalizing Small Molecules. ACS Infect. Dis..

[29] Ghosh S., Dellibovi-Ragheb T.A., Kerviel A., Pak E., Qiu Q., Fisher M., Takvorian P.M., Bleck C., Hsu V.W., Fehr A.R. (2020). β-Coronaviruses Use Lysosomes for Egress Instead of the Biosynthetic Secretory Pathway. Cell.

[30] Hofmann I., Munro S. (2006). An N-Terminally Acetylated Arf-like GTPase Is Localised to Lysosomes and Affects Their Motility. J. Cell Sci..

[31] Ponsford A.H., Ryan T.A., Raimondi A., Cocucci E., Wycislo S.A., Fröhlich F., Swan L.E., Stagi M. (2021). Live Imaging of Intra-Lysosome PH in Cell Lines and Primary Neuronal Culture Using a Novel Genetically Encoded Biosensor. Autophagy.

[32] Koepke L., Hirschenberger M., Hayn M., Kirchhoff F., Sparrer K.M. (2021). Manipulation of Autophagy by SARS-CoV-2 Proteins. Autophagy.

[33] Bestion E., Jilkova Z.M., Mège J.-L., Novello M., Kurma K., Pour S.T.A., Lalmanach G., Vanderlynden L., Fizanne L., Bassissi F. (2020). GNS561 Acts as a Potent Anti-Fibrotic and pro-Fibrolytic Agent in Liver Fibrosis through TGF-Β1 Inhibition. Ther. Adv. Chronic. Dis..

[34] Zhao M.-M., Yang W.-L., Yang F.-Y., Zhang L., Huang W.-J., Hou W., Fan C.-F., Jin R.-H., Feng Y.-M., Wang Y.-C. (2021). Cathepsin L Plays a Key Role in SARS-CoV-2 Infection in Humans and Humanized Mice and Is a Promising Target for New Drug Development. Sig. Transduct. Target Ther..

[35] Hashimoto R., Sakamoto A., Deguchi S., Yi R., Sano E., Hotta A., Takahashi K., Yamanaka S., Takayama K. (2021). Dual Inhibition of TMPRSS2 and Cathepsin B Prevents SARS-CoV-2 Infection in IPS Cells. Mol. Ther. Nucleic Acids.

[36] Rosa R.B., Dantas W.M., do Nascimento J.C.F., da Silva M.V., de Oliveira R.N., Pena L.J. (2021). In Vitro and In Vivo Models for Studying SARS-CoV-2, the Etiological Agent Responsible for COVID-19 Pandemic. Viruses.

[37] Dittmar M., Lee J.S., Whig K., Segrist E., Li M., Kamalia B., Castellana L., Ayyanathan K., Cardenas-Diaz F.L., Morrisey E.E. (2021). Drug Repurposing Screens Reveal Cell-Type-Specific Entry Pathways and FDA-Approved Drugs Active against SARS-Cov-2. Cell Rep..

[38] Pruijssers A.J., George A.S., Schäfer A., Leist S.R., Gralinksi L.E., Dinnon K.H., Yount B.L., Agostini M.L., Stevens L.J., Chappell J.D. (2020). Remdesivir Potently Inhibits SARS-CoV-2 in Human Lung Cells and Chimeric SARS-CoV Expressing the SARS-CoV-2 RNA Polymerase in Mice. bioRxiv.

[39] Srinivasa A., Tosounidou S., Gordon C. (2017). Increased Incidence of Gastrointestinal Side Effects in Patients Taking Hydroxychloroquine: A Brand-Related Issue?. J. Rheumatol..

[40] Mavrikakis M., Papazoglou S., Sfikakis P.P., Vaiopoulos G., Rougas K. (1996). Retinal Toxicity in Long Term Hydroxychloroquine Treatment. Ann. Rheum. Dis..

[41] Easterbrook M. (1993). The Ocular Safety of Hydroxychloroquine. Semin. Arthritis Rheum..

[42] Iglesias Cubero G., Rodriguez Reguero J.J., Rojo Ortega J.M. (1993). Restrictive Cardiomyopathy Caused by Chloroquine. Br. Heart J..

[43] Costedoat-Chalumeau N., Hulot J.-S., Amoura Z., Leroux G., Lechat P., Funck-Brentano C., Piette J.-C. (2007). Heart Conduction Disorders Related to Antimalarials Toxicity: An Analysis of Electrocardiograms in 85 Patients Treated with Hydroxychloroquine for Connective Tissue Diseases. Rheumatology.

[44] Genoscience Pharma Phase 1/2a Study to Evaluate the Safety, Activity, and Pharmacokinetics of Escalating Doses of GNS561 in Patients With Primary and Secondary Liver Cancer. clinicaltrials.gov.

[45] Harding J.J., Awada A., Decaens T., Roth G., Merle P., Kotecki N., Dreyer C., Ansaldi C., Rachid M., Mezouar S. (2021). First-in-Human Phase I, Pharmacokinetic (PK), and Pharmacodynamic (PD) Study of Oral GNS561, a Palmitoyl-Protein Thioesterase 1 (PPT1) Inhibitor, in Patients with Primary and Secondary Liver Malignancies. JCO.

