# Supplementary material for: GNS561 Exhibits Potent Antiviral Activity against SARS-CoV-2 through Autophagy Inhibition
Source: Viruses. 2022 Jan 12;14(1):132. doi: 10.3390/v14010132 (PMC8778678; doi:10.3390/v14010132)
Supplement: Supplementary file 1 [file viruses-14-00132-s001.zip › viruses-1498580-supplementary.pdf]

Supplementary material

GNS561 exhibits potent antiviral activity against SARS-CoV-2 through autophagy inhibition

Eloïne Bestion[1,2,3], Keivan Zandi[4], Sandrine Belouzard[5], Julien Andreani[2,3], Hubert Lepidi[6], Marie Novello[1], Clara Rouquairol[1], Jean-Pierre Baudoin[2,3], Madani Rachid[1], Bernard La Scola[2,3], Jean-Louis Mege[2,3], Jean Dubuisson[5], Raymond F. Schinazi[4,†], Soraya Mezouar[1,2,3, †]and Philippe Halfon[1,*,†]

Tables

Table S1. Primer and probe sequences for SARS-CoV-2 E (cells) and N (mouse) genes q-RTPCR

investigation

SARS-CoV-2 Primers/Probes		Sequences
**Cell assays**	Forward	5'-GAC CCC AAA ATC AGC GAA AT-3'
	Reverse	5'-TCT GGT TAC TGC CAG TTG AAT CTG-3'
	Probe	5'-FAM-ACC CCG CAT TAC GTT TGG TGG ACC-BHQ1-3'
**Mouse assays**	Forward	5'-TAA TGG ACC CCA AAA TCA GC-3'
	Reverse	5'-GAA TCT GAG GGT CCA CCA AA-3'

Figures

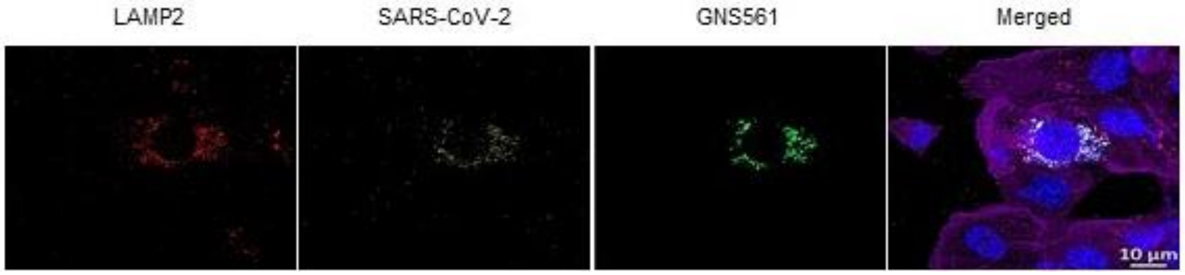

Figure S1. SARS-CoV-2 and GNS561 localization inside lysosomes. After 2 hours of treatment with 4 µM GNS561, Vero E6 cells were infected with SARS-CoV-2 IHUMI-6 strain for an additional 48 hours. Representative confocal images showing the localization of SARS-CoV-2 (yellow) inside LAMP2-positive lysosomes (red) together with GNS561 (green) inside Vero E6 cells are exposed.

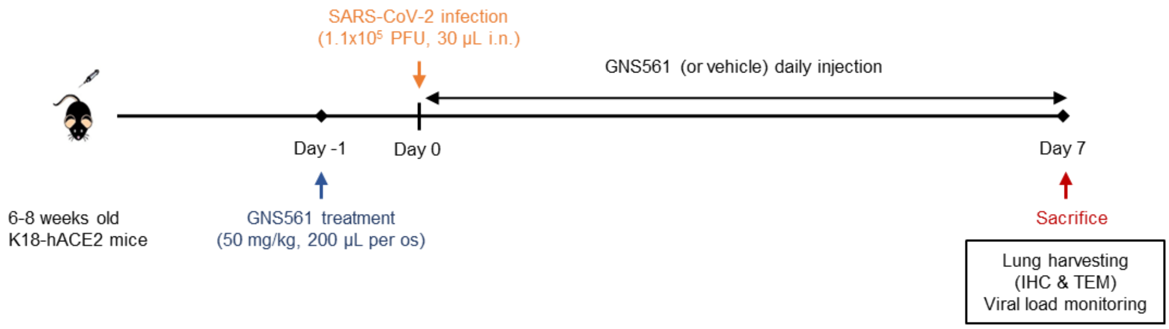

Figure S2. Study design of K18-hACE C57BL/6J mice model. Eight to nine-week-old K18-hACE C57BL/6J mice were treated with GNS561 (50 mg / kg, 200 µL) or with vehicle per os 24h before intranasally SARS-CoV-2 infection (1.1x105 PFU, 30 µL). Mice were then daily injected with GNS561 or vehicle until sacrifice 7 days post-infection. IHC: immunohistochemistry, i.n.: intranasally, TEM: transmitted electron microcopy.
